# G6PD Variants and Haemolytic Sensitivity to Primaquine and Other Drugs

**DOI:** 10.3389/fphar.2021.638885

**Published:** 2021-03-15

**Authors:** Germana Bancone, Cindy S. Chu

**Affiliations:** ^1^ Shoklo Malaria Research Unit, Mahidol-Oxford Tropical Medicine Research Unit, Faculty of Tropical Medicine, Mahidol University, Mae Sot, Thailand; ^2^ Centre for Tropical Medicine and Global Health, Nuffield Department of Medicine, University of Oxford, Oxford, United Kingdom

**Keywords:** haemolysis, G6PD (glucose-6-phosphate dehydrogenase), deficiency, primaquine, AHA, genotyping, phenotype

## Abstract

Restrictions on the cultivation and ingestion of fava beans were first reported as early as the fifth century BC. Not until the late 19th century were clinical descriptions of fava-induced disease reported and soon after characterised as “favism” in the early 20th century. It is now well known that favism as well as drug-induced haemolysis is caused by a deficiency of the glucose-6-phosphate dehydrogenase (G6PD) enzyme, one of the most common enzyme deficiency in humans. Interest about the interaction between G6PD deficiency and therapeutics has increased recently because mass treatment with oxidative 8-aminoquinolines is necessary for malaria elimination. Historically, assessments of haemolytic risk have focused on the clinical outcomes (e.g., haemolysis) associated with either a simplified phenotypic G6PD characterisation (deficient or normal) or an ill-fitting classification of G6PD genetic variants. It is increasingly apparent that detailed knowledge of both aspects is required for a complete understanding of haemolytic risk. While more attention has been devoted recently to better phenotypic characterisation of G6PD activity (including the development of new point-of care tests), the classification of G6PD variants should be revised to be clinically useful in malaria eliminating countries and in populations with prevalent G6PD deficiency. The scope of this work is to summarize available literature on drug-induced haemolysis among individuals with different G6PD variants and to highlight knowledge gaps that could be filled with further clinical and laboratory research.

## Introduction

The first clinical descriptions of what we now know as drug-induced haemolysis were documented in the late 19th century (Mulè-Bertolo, 1886). Cases presenting with jaundice and haemoglobinuria after exposure to raw fava beans (ingested or inhaled) were observed in the Mediterranean region (Meletis and Konstantopoulos, 2004; Khazaei et al., 2019). By the early 20th century this clinical syndrome was known as “favism.” A male predominance and familial patterns were noted (Gasbarrini, 1915). The biochemical cause of favism remained unknown until the mid-20th century when a similar clinical syndrome of acute haemolytic anaemia (AHA) was observed in ethnically African males who received primaquine for the treatment of *Plasmodium vivax* malaria (Hockwald et al., 1952). Large haemoglobin drops were observed during the first week of primaquine dosing with subsequent haematologic recovery beginning the following week (Beutler et al., 1954; Dern et al., 1954). These male individuals were given a clinical diagnosis of “primaquine sensitivity” or having “primaquine sensitive” erythrocytes. Blood films showed a pattern of intravascular haemolysis with Heinz bodies visualized in red blood cells (Dern et al., 1955). Like in favism, jaundice and haemoglobinuria were observed. A similarity between the haemolytic anaemia caused by “favism,” plasmoquin (the 8-aminoquinoline precursor to primaquine) and sulfa drugs had been recognized previously (Rachmilewitz et al., 1945). Further assessments showed that these haemolytic syndromes indeed shared the same biochemical defect; a deficiency in the glucose-6-phosphate dehydrogenase (G6PD) enzyme (Carson et al., 1956). As data from these two syndromes converged they enabled scientists to determine the X-linked nature of the disorder (Childs et al., 1958; Zinkham et al., 1958). Primaquine sensitivity was also noted in females, but the clinical syndrome was different from males. Rather than two distinct populations of subjects who were either primaquine sensitive or not, in females the haemolysis ranged from asymptomatic individuals with abnormal laboratory values which was not observed in males, to AHA as observed in males (Tarlov et al., 1962).

In this review we summarise the available data on drug-induced haemolysis accumulated in the last 70 years with a specific focus on the G6PD variants studied. Limitations of current laboratory and clinical characterisation as well as possible future research approaches are discussed.

## Methodology

A research was conducted into PubMed using the words “G6PD,” “drug” and “haemolysis.” The purpose of the search was to find published literature reporting cases or original clinical studies where haemolysis was investigated after use of drugs in G6PD deficient subjects. Studies where drugs were used *in vitro* on cultured cells or in subjects transfused with G6PD deficient RBCs were excluded. Since the majority of studies and case reports concerned treatment with primaquine and other 8-aminiquinolines which are already reported in the main text, Supplementary Table S1 only includes literature on drugs other than 8-aminoquinolines.

## Diagnosis of G6PD Deficiency

G6PD is the first enzyme of the pentose phosphate pathway (or hexose monophosphate shunt, HMS), the rate-limiting step for the production of reducing factor NADPH needed in cellular anti-oxidant response. G6PD is a highly conserved housekeeping gene across species, (Kletzien et al., 1994), that shows a remarkable variability in humans with over 200 polymorphic variants, mostly single nucleotide mutations, described so far (Gomez-Manzo et al., 2016). Mutations on the G6PD gene are associated with different protein defects causing variable degrees of decreased enzymatic activity and, consequentially, decreased capacity to respond to cellular oxidative challenges. This is particularly evident in red blood cells where the HMS is the only source of NADPH and oxidative stress in G6PD deficient cells can cause haemolysis. Mutations that cause complete loss of function are not compatible with life. As an X-linked trait, males express a clear phenotype of enzymatic deficiency when carrying the mutation and a clear haemolytic sensitivity in most of their red blood cells. In females, one X chromosome undergoes inactivation in each cell through the lyonization process in the early embryonic stage. In homozygous mutated females, a similar phenotype to that observed in deficient males is observed. In heterozygous females, because of different X-chromosome inactivation patterns, a large variability in phenotypes is observed. Since only RBCs expressing the mutated protein are susceptible to haemolysis, the haemolytic risk in heterozygous women is also variable and cannot be predicted by genotype alone.

It is not surprising therefore that different approaches can be used to diagnose G6PD deficiency at the genotypic and phenotypic level; since none of them can be considered completely satisfactory, the choice of tests needs to be based on the clinical interest or on the research question. If the aim is to assess the prevalence and characterize the molecular variation at the population level, genotyping is sufficient. There is a large amount of knowledge accumulated on geographical variability of G6PD (Howes et al., 2013; Bancone et al., 2019; Lippi and Mattiuzzi, 2020; Zheng et al., 2020) so that genotyping can be performed on a small panel of known mutations already described in a given population. Furthermore, full gene sequence can be used to explore rarer variants or to investigate populations lacking prior information. This approach is greatly simplified (and less expensive) when genotyping is performed only among subjects who show phenotypic deficiency.

If the aim is to treat with oxidative drugs or to assess haemolytic risk, a phenotypic test can be sufficient to identify subjects or patients with the appropriate levels of enzyme and initiate clinical management, without a genotypic confirmatory test. G6PD phenotypes can be assessed in numerous ways; historically protein analysis (including electrophoretic mobility, Michaelis constants for G6P, relative rate of utilization of 2-deoxy G6P, Km for NADP and Ki for NADPH, pH and thermal stability) were always used together with assessment of enzymatic activity to characterize the variants (WHO Scientific Group, 1967; Vives-Corrons et al., 1990; Kotaka et al., 2005; Satyagraha et al., 2015; Boonyuen et al., 2016). Currently the gold standard spectrophotometric assay is the most commonly used laboratory test to characterize G6PD enzymatic activity. The test is based on changes of NADPH over time in hemolysates of blood incubated with the substrate; it gives a result of G6PD activity normalized by grams of haemoglobin (or number of RBCs). Cytochemical techniques with flow-cytometric read out (Van Noorden and Vogels, 1985; Shah et al., 2012) detects the presence of active enzyme at the level of single RBCs, including in younger RBCs of deficient subjects and, in heterozygous women, in the RBC population where the mutated chromosome has been inactivated. The use if these techniques allows a more detailed characterisation of blood samples in the presence of anaemia and reticulocytosis (Bancone et al., 2017). While bringing highly correlated results, spectrophotometric and flow-cytometric analyses provide somehow complementary information that can be used to follow the progression of haemoglobin drop and recovery during an haemolytic event and to assess the independent contribution of both reticulocytes and mature RBCs to the total enzymatic activity.

New point-of-care (POC) quantitative and semi-quantitative G6PD tests, equivalent to the spectrophotometric assay, have been developed and at least one is currently available and validated in adult blood (Pal et al., 2019). A major difference between these quantitative tests and rapid phenotypic tests used for screening lies on the outcome as a qualitative result (i.e. binary: deficient or normal) based on an enzymatic activity threshold of ca 30–40% of normal. Qualitative tests such as the fluorescent spot test (FST, (Beutler and Mitchell, 1968)) and RDTs (Ley et al., 2019) provide a diagnosis of G6PD deficiency on samples that have an enzymatic activity lower than this threshold. They can be powerful screening tools and are useful in identifying subjects at higher risk of drug-induced haemolysis, but they cannot be used to identify heterozygous women with intermediate enzymatic activity who might still experience drug-induced haemoglobin drops.

In summary, genotyping can be used reliably for epidemiologic and anthropologic research, and being increasingly simple and cheap allows batching of samples and analysis of very large sample sizes. Due to phenotypic variability of heterozygote females and limited knowledge of haemolytic risk associated with specific variants (see below), genotyping cannot be used alone for clinical management. Characterisation of phenotype is more complicated, has lower throughput, and requires freshly collected blood. Furthermore, results depend from the type of test used. The quantitative phenotype is certainly most informative for clinical management and for assessment of the haemolytic risk of a certain drug regimen in persons with abnormal G6PD activity.

## The Current Classification of G6PD Variants and Haemolytic Risk

In 1989, The “WHO classification” (WHO Working Group, 1989) reported a list of known variants with associated enzymatic phenotypes based on a previous list compiled by Yoshida in 1971 (WHO Scientific Group, 1967; Yoshida et al., 1971). The classification included variants that were reported and described often in a single subject, and analysed with different laboratory techniques. Over time, with better genotyping techniques, different variants have been found to be caused by the same DNA mutation. A clear limitation on this phenotypic classification was the lack of protein characteristics that could be correlated unequivocally to *in vivo* haemolytic susceptibility (Yoshida, 1973; Yoshida, 1980). The WHO report indeed stated that “The spectrum of clinical manifestations associated with individual G6PD-deficient variants should be defined further.” Over the years, little clinical data have been produced to link the laboratory characterisation with the haemolytic risk during oxidative stress (e.g., drug exposure), yet the classification has been used very often to indicate a graded haemolytic risk associated with the different classes of G6PD variants.

Variants in WHO class I which are found sporadically and usually discovered in subjects with chronic non-spherocytic haemolytic anaemia (CNSHA), would be expected to have the best characterized phenotype. A review in 2000 by Fiorelli showed that a low inhibition constant (Ki) for NADPH, a higher Km for substrates and a reduced thermostability were common biochemical features among these mutations (Fiorelli et al., 2000); furthermore, they were mostly found in exon 10 and showed usually, but not always, severely decreased enzymatic activity of <10% of normal in RBCs and sometimes reduced enzymatic activity in white blood cells (Gray et al., 1973). While most subjects with CNSHA variants develop neonatal hyperbilirubinaemia with increased reticulocyte counts, not all have anaemia or require transfusion after a haemolytic event.

Classification of variants into class II and III is probably even more controversial since data accumulated over the years have shown an extremely large variability in protein features and very little differences in terms of residual enzymatic activity. Thus, the main characteristic (enzymatic activity) used to discriminate between the two classes seems insufficient for a precise categorisation (Luzzatto, 2009) of haemolytic risk.

The WHO classification has also been misinterpreted at times and used as a phenotypic classification of “individuals” rather than a categorisation of residual enzymatic activities observed in hemizygous males with the specific variant (Kim et al., 2011).

Most recently a number of *in-silico* analyses have been published showing predictions of the enzymatic defect solely based on genotypic data and the WHO classification (Wiwanitkit, 2005; Chiu et al., 2019). Some of these *in-silico* studies have used unreliable data of enzymatic activity assessed either during a haemolytic event or shortly after (Lee et al., 2017); in one study, the enzymatic phenotype was assessed on heterozygous women (Chaowanathikhom et al., 2017). These *in-silico* predictions need to be supported by better phenotypic and clinical characterizations to be meaningful for clinical practice.

## G6PD Variants That Have Been Associated With Drug Induced Haemolysis

More than 70 years have passed since the first description of G6PD deficiency. The evidence accumulated over time has been produced by several clinical trials using different rationales, methodologies and laboratory techniques. Typically, the older published literature have reported detailed haematologic and clinical data with none or very limited genetic characterisation while more recent studies have provided detailed molecular analyses using modern technology but more limited phenotypic and clinical data. In clinical trials with known haemolytic agents, G6PD deficient patients have been excluded purposively while in many case reports the G6PD genotype has not been analysed or documented (Supplementary Table S1).

Inter-individual variability on metabolisation of prodrugs (including primaquine) into their active metabolites by hepatic cytochromes P450 is a relatively recent discovery (Watkins, 1990); analysis of drug concentration in blood is often required in order to assess the actual drug exposure. For primaquine, CYP2D6 would need to be characterized to assess haemolytic risk together with the G6PD variants (Pybus et al., 2013).

Therefore, direct comparisons in haemolytic response between specific G6PD variants were not made here as an incomplete knowledge of all factors involved could potentially result in misleading conclusions.

The first observations of AHA caused by pamaquine, the first synthetic 8-aminoquinoline, were reported in non-Caucasians of African, Indian, and Chinese descent. Mutations were not characterised but notably they were not associated with blackwater fever (massive haematuria caused by malaria related intravascular haemolysis) or risk for blackwater fever (Earle et al., 1948; Hardgrove and Applebaum, 1946). The largest review of drug-induced haemolysis to date analysed all published haemolytic events associated with 8-aminoquinoline use and found a low overall mortality (Recht et al., 2014).

### G6PD*A-

The G6PD variant with the most detailed data is the A- (202G > A and 376A > G) which is common in Africa and persons of African descent (especially in the Americas). The 376A > G mutation identifies the A variant on which background the A- variant (with the additional 202G > A mutation) is thought to have arisen (Yoshida and Takizawa, 1988). The variant is most often identified in the laboratory by only testing the 202G > A mutation but several studies have demonstrated a greater heterogeneity of genotypes in African populations (Jalloh et al., 2008; Clark et al., 2009). Therefore, what has been historically considered “A- phenotype” in African and Afro-American patients might be composed of different variants with possibly different residual enzymatic activity (Beutler et al., 1989) including 680G > T (Hirono and Beutler, 1988), 542A > T (Santa Maria, (Clark et al., 2009)), 968C > T (Betica Selma, (Clark et al., 2009)), or 311G > A (Sierra Leone, (Jalloh et al., 2008)) additional mutations. The associated phenotype of each of these different A- variants has not been described; indeed some of the largest population studies in Africa where G6PD deficiency has been characterised after the advent of modern molecular techniques, have not documented phenotypes in almost 30,000 malaria patients and controls (Rockett et al., 2014). The lack of phenotypic characterisation has limited our ability to know the potential clinical response a given G6PD variant has to an oxidative insult.

#### Primaquine for *Plasmodium Vivax* Radical Cure

**FIGURE 1:**
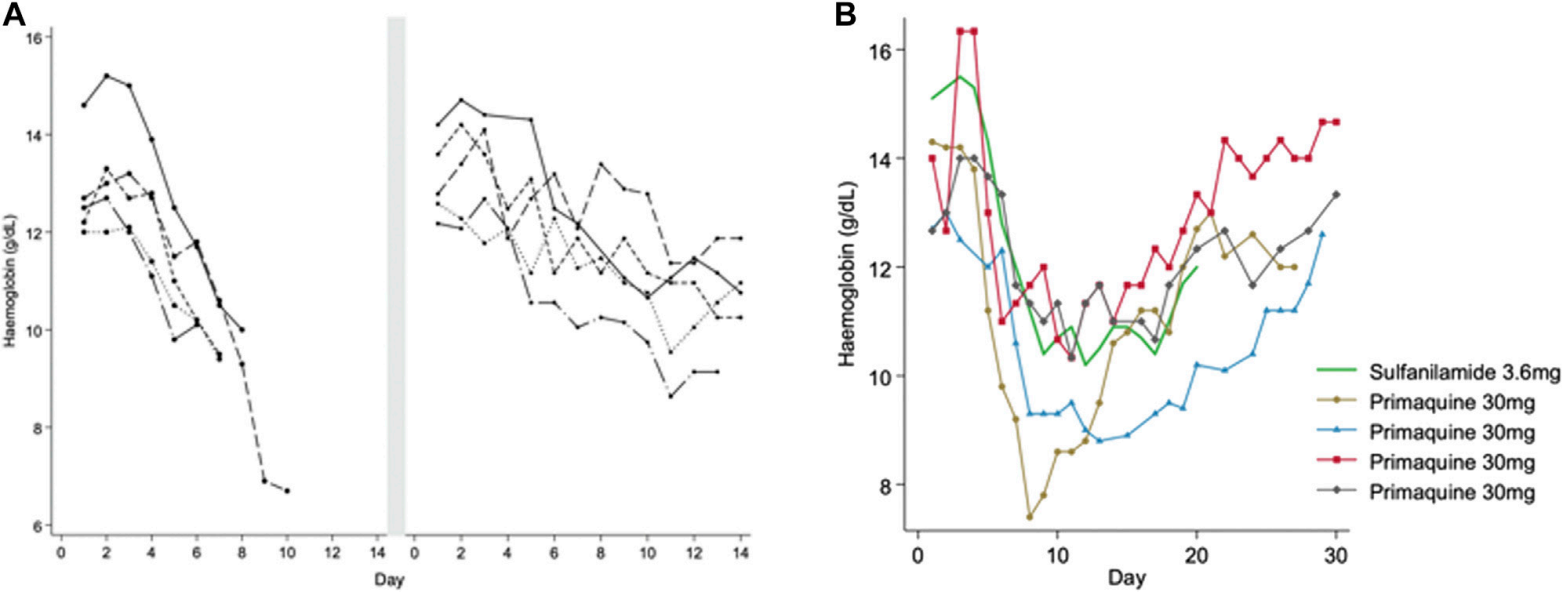
**(A,B)** Haemolytic and recovery responses to high (adult dose 30 mg) and low (adult dose 15 mg) daily primaquine doses in males with presumed G6PD*A- deficiency **(A)** is replotted from Hockwald et al. (1952). This figure shows haemoglobin values in five healthy G6PD deficient males that developed acute haemolytic anaemia when taking PQ 30 mg daily. Dosing was stopped early before completing the 14-day course. After haemoglobin recovery to steady state (duration of time not reported) the same subjects were given PQ 15 mg daily for 14 days. The smaller symbol size represents the low (15 mg) daily PQ dose. Figure 1B is replotted from Dern et al. (1954) and Dern et al. (1955), and Kellermeyer et al. (1961). This figure shows haemoglobin values in healthy G6PD deficient males receiving continuous daily doses of primaquine (PQ) 30 mg (black) and sulfanilamide 3.6 mg (green). The lines end when dosing was stopped, except in one person continuing to day 50 (triangle symbol), see Figure 2. The symbols identify the same subjects in Figure 2.

In one of the earliest primaquine trials (Hockwald et al., 1952), varying regimens of primaquine were given to 110 healthy men of African descent (presumably with A- mutation): primaquine 30 mg daily for 14 days (a high dose regimen currently recommended in parts of the Asia-Pacific region) with and without quinine or chloroquine. The regimens were given in daily or divided doses each day. Five men developed severe anaemia, which was defined as a “decrease in haemoglobin which necessitated discontinuation of the drug before 14 days.” Moderate anaemia was defined as a haemoglobin drop of more than 4 g/dl during primaquine administration. All five men had elevated bilirubin and four of them developed haemoglobinuria. To understand whether or not haemolysis would occur at a lower primaquine dose, a challenge with primaquine 15 mg daily for 14 days (a low dose regimen which is now the most common globally) was given in the same subjects after they reached their steady state haemoglobin. At this dose a milder haemolysis occurred (Figure 1A) and it was concluded that the 15 mg primaquine regimen could be given without special medical supervision in persons of African descent. However, the timing of the primaquine 15 mg challenge at steady state was not reported; if it was given less than 3–4 months after the initial haemolysis, the resulting haemolytic response may not have reflected the change from their true steady state. Subsequent studies in subjects of African descent assessed the haemolytic effect of primaquine with prolonged daily regimens and with dose increases at varying timepoints after recovery from the initial haemolysis. When a daily 30 mg primaquine dose was continued through the haemolysis there was progression to a “recovery phase” and haemoglobin increased even when the same dose of primaquine was continued through the “recovery phase” (Dern et al., 1954) (Figure 1B). If dosing was prolonged (e.g., for months) the haemoglobin returned to pre-dosing levels (presumed steady state) and remained in an “equilibrium phase,” leading the authors to conclude that the haemolytic effect was self-limited. In red cell labelling studies, it was determined that primaquine 30 mg daily caused older red blood cells (RBC) to haemolyse whilst RBCs that were 8–21 days old (young RBCs) did not haemolyse (Beutler et al., 1954). Thus, it became clear that there was a different response to primaquine between old and young RBCs.

**FIGURE 2:**
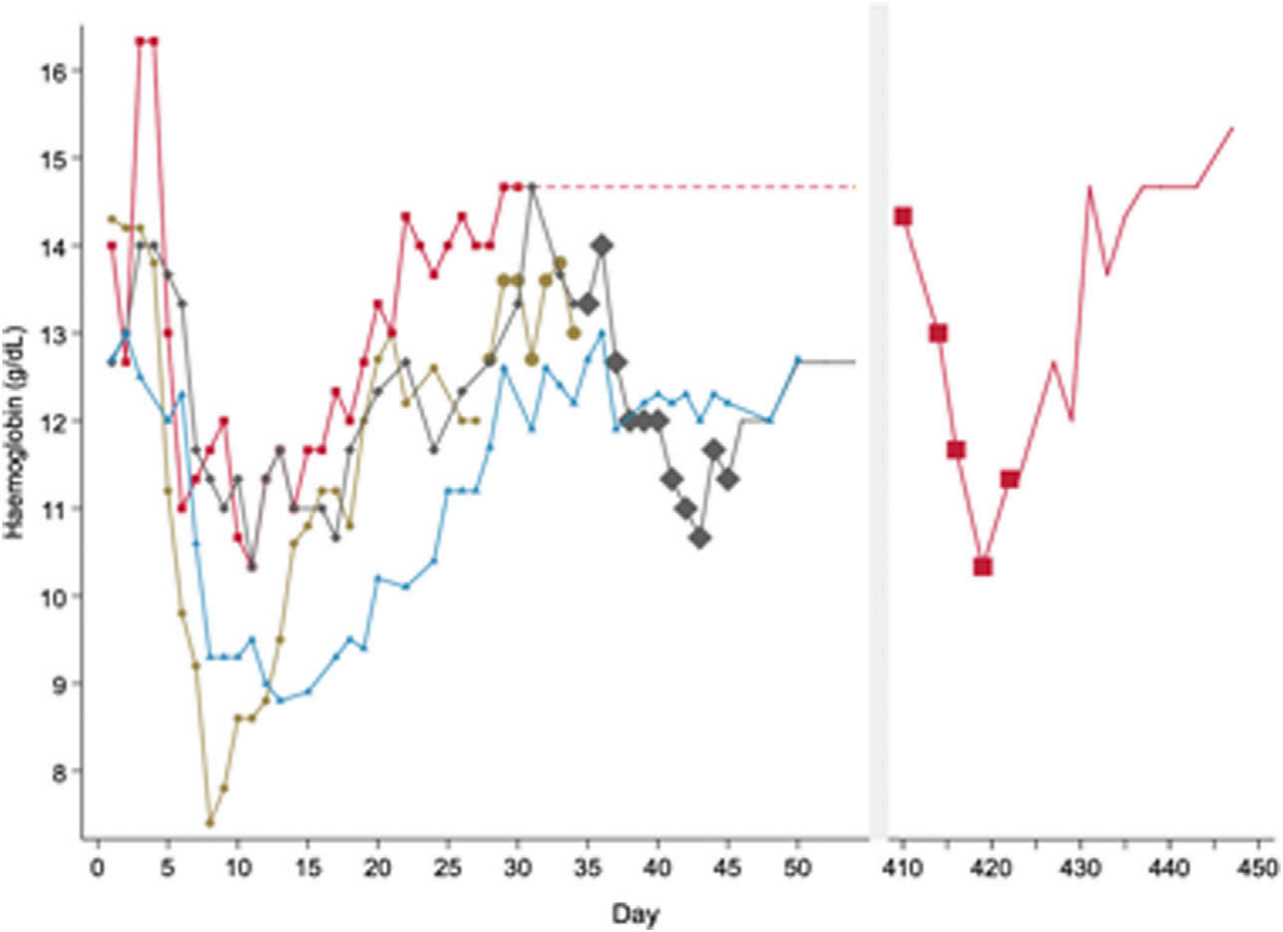
Pattern of haemolysis in healthy male subjects with presumed G6PD*A- deficiency when exposed to different doses of primaquine of varying durations. In this figure, subjects express a haemolytic, recovery and equilibrium phase when taking primaquine (PQ) 30 mg daily. Haemolysis occurs during the equilibrium phase if primaquine doses are increased. This figure is replotted from raw data in healthy males published in (Dern et al., 1955) and (Kellermeyer et al., 1961). These are the same subjects as represented in Figure 1A. Each line represents one subject. The symbol size increases to reflect PQ dose; no symbol means no PQ was given. All subjects started PQ 30 mg daily. One subject (red) received PQ 30 mg daily for over one year until the dose was increased to PQ 240 mg daily from days 410 to 422. Another subject (grey) received PQ 240 mg from days 35 to 45. A third subject (brown) received PQ 60 mg daily from days 28 to 34. The fourth subject (blue) did not have any dose increases and continued PQ for 50 days.

After the enzymatic defect was identified as G6PD deficiency, scientists sought to assess whether RBCs had an all or none effect, or if there was an age-related gradient of G6PD activity. To do this, primaquine was increased up to 240 mg daily in both the recovery and equilibrium phases after healthy males had been already receiving 30 mg primaquine daily. A second haemolysis was observed in both phases (Figure 2). These data suggested that the “recovery phase” was a result of compensated bone marrow RBC production with G6PD replete reticulocytes and primaquine induced haemolysis was dose dependent (Kellermeyer et al., 1961).

#### Weekly and Single Dose Primaquine

The physiologic response and resistance to haemolysis was also evident for the weekly 0.75 mg/kg dose (approximates a 45 mg adult dose), a dose that was assessed for causal prophylaxis and radical cure. The 45 mg weekly primaquine dose caused less haemolysis in a male of African descent with known primaquine sensitivity when compared to primaquine 60 mg (approximates a 0.5 mg/kg dose) and 30 mg (approximates a 0.25 mg/kg dose) daily doses, and a 60 mg weekly dose for 8 weeks. These four dosing regimens were given in the same individual at 6-month intervals (Alving et al., 1960). After this study, there are no published haematologic observations and outcomes for the weekly primaquine dosing regimen in G6PD deficient patients until nearly 50 years later (*see* Mediterranean variant, (Leslie et al., 2008)).

Dose dependent primaquine haemolysis has been observed also in individuals with intermediate G6PD activity. In studies conducted in the late 1950s, females with intermediate G6PD activity (identified by the glutathione stability test) of African descent (presumably heterozygous for G6PD A-) were shown to develop haemolysis after taking primaquine. These were the first observations of the widely variable primaquine-induced haemolysis in females, with haemoglobin declines ranging from asymptomatic to AHA (Alving et al., 1958). Many decades would pass before the pattern of haemolysis after primaquine in G6PD heterozygous females would be described in other G6PD variants.

Indeed, the dose dependent nature of drug induced haemolysis has been verified in *Plasmodium falciparum* transmission blocking studies evaluating the safety of single dose primaquine. The doses assessed ranged from 0.25 to 0.75 mg/kg and higher single doses of primaquine caused larger haemoglobin drops but no severe haemoglobin or haematocrit drops were observed. Single low-dose PQ (0.25 or 0.4 mg/kg) in combination with AL and DP was associated with mild and transient drops in haemoglobin in healthy adult volunteers in Burkina Faso (Bastiaens et al., 2018). In a cohort of Ugandan children with uncomplicated *P. falciparum* malaria and a normal test result by FST, who had G6PD mutations (including homo and hemizygous A-), drops in haemoglobin concentrations were observed after a single dose of 0.75 or 0.4 mg/kg but not 0.1 mg/kg. Drops in haemoglobin were transient, with no participant experiencing clinical symptoms suggestive of anaemia (Eziefula et al., 2014).

In Mali, malaria negative G6PD deficient adult males received 0.40, 0.45, or 0.50 mg/kg of single dose PQ with no evidence of symptomatic haemolysis and only had mild and uncommon adverse events considered related to the study drug (Chen et al., 2018). Of note, only the 202G > A mutation was tested in the study but other investigations in Mali demonstrated that other deficient genotypes are common in the country (Maiga et al., 2014).

#### Other Drugs

Together with antimalarial primaquine, the haemolytic profile of chlorproguanil dapsone artesunate (CDA) given to patients with A- variant has been extensively described. At a regimen of 2.0 mg/kg/d of chlorproguanil, 2.5 mg/kg/d dapsone and 4 mg/kg/d artesunate given for 3 days, Rwandan children with uncomplicated *P. falciparum* malaria with G6PD A- had significantly lower haematocrit as compared to wild types until day 7 (Fanello et al., 2008). Haematologic recovery began the following week resulting in complete recovery and no difference in haematocrit compared to wild types at day 14. With the same dose tested in seven African countries, occurrences of a composite haemoglobin safety endpoint (haemoglobin drop ≥4g/dL or ≥40% vs. baseline, haemoglobin <5g/dL, or blood transfusion) were significantly higher in G6PD A- hemi and homozygous females as compared to G6PD normal patients (Tiono et al., 2009). In a trial to test four ACTs [dihydroartemisinin-piperaquine (DP), amodiaquine-artesunate (AQ + AS), artemether-lumefantrine (AL), and CDA] in Uganda and Mozambique, malaria paediatric patients with G6PD A- (hemi and homozygotes) treated with CDA had non-significant higher odds of experiencing a haemoglobin drop ≥2 g/dl within the first four days after treatment (Van Malderen et al., 2012). Safety in G6PD A- infants was assessed during a trial conducted in infants to test three antimalarial regimens for Intermittent Preventive Treatment in moderate and low transmission sites in northeast Tanzania. At day 7, homozygous and hemizygous genotypes were associated with a higher odds of having a haemoglobin level <8 g/dl and greater absolute reductions in haemoglobin in all the regimens combined (Poirot et al., 2015).

When Methylene Blue (MB) was used against malaria, significant haemoglobin drops in A- G6PD hemizygous and homozygous children were shown in West Africa using the gametocytocidal dose (15 mg/Kg per day for 3 days) but with limited clinical impact (Meissner et al., 2005; Muller et al., 2013). No haemolysis was observed in G6PD deficient adults from Burkina Faso (presumably with the A- variant) treated with 780 mg MB over 3 days (Mandi et al., 2005).

Early observation of haemolytic and recovery response similar to primaquine were recorded with other compounds, such as sulfonamides (Dowling and Lepper, 1943) and nitrofurans (West and Zimmerman, 1956; Kimbro et al., 1957).

Treatment with acetylsalicylic acid, sulfonamide or single low dose dapsone has not shown to cause AHA in adults with A- variant (Degowin et al., 1966; Norden et al., 1968; Glader, 1976). Single case reports of AHA have been published on proparacetamol and glibenclamide (Oliver et al., 2001; Vinzio et al., 2004). Use of pegloticase for treatment of refractory gout has been showed to be associated with AHA in a Honduran patient with confirmed A- mutations (Geraldino-Pardilla et al., 2014) and in two African American males with presumably the same mutation (Owens et al., 2016; Adashek and Bourji, 2018).

### G6PD*Mediterranean

The Mediterranean mutation (563C > T) is common in Southern Europe and the Middle-East and presumed to be the cause of early description of favism in Italy and Greece. While the most predominant, the Mediterranean variant represents only 60–80% of all mutations in those countries (Martinez di Montemuros et al., 1997; Menounos et al., 2000) and this heterogeneity has been recognized only in the early 1970s (Stamatoyannopoulos et al., 1971). In Afghanistan, over 95% of G6PD deficient males harboured the Mediterranean mutation (Leslie et al., 2013).

#### Primaquine

A Sardinian healthy male with presumed Mediterranean variant was treated with 30mg primaquine daily which caused non self-limiting AHA (Pannacciulli et al., 1965). Efficacy of the weekly PQ dose was evaluated in Afghan refugees in Pakistan and one G6PD deficient male (variant not reported but presumably Mediterranean) was enrolled; No serious adverse events were noted (Leslie et al., 2008). In Bangladesh, a 9 year old boy with *P. vivax* experienced AHA after overdosing on 7.5mg daily dose PQ (equivalent to a 4.8mg/kg total dose) for radical cure (Phru et al., 2020).

#### Other Drugs

In confirmed G6PD Mediterranean subjects, one child with the mutation taking aspirin (100 mg/kg/d for 10 days) for systemic arthritis in Italy (Meloni et al., 1989) had AHA. However, no AHA was found in series of five patients with G6PD Mediterranean and Seattle treated with the same dose (Biscaglia et al., 2015). A case report after use of ciprofloxacin was reported in an adult patient with Mediterranean mutation (Sansone et al., 2010). Use of feprazone against fever in children did not cause AHA (Meloni et al., 1982). In cases where Mediterranean variant was presumed (no test result reported), subsequent case reports of AHA have been described with chlorproguanil-dapsone (Leslie et al., 2007), dapsone alone (Murphy and Grossman, 2016), normal or excessive doses of paracetamol (Bartsocas et al., 1982; Phillpotts et al., 2014) and other uncommonly used drugs (Supplementary Table S1).

### G6PD*Mahidol, Viangchan and Other Asian Variants

The Mahidol (487G > A) variant is commonly found in Myanmar and western Thailand (Laosombat et al., 2005; Bancone et al., 2014). The Viangchan variant is the most prevalent in Thailand, Lao PDR, Vietnam and Cambodia (Bancone et al., 2019); Union, Canton, Kaiping and Coimbra are found at a low prevalence in most East- and SouthAsian countries (Howes et al., 2013).

#### Primaquine for *Plasmodium vivax* Radical Cure

**FIGURE 3:**
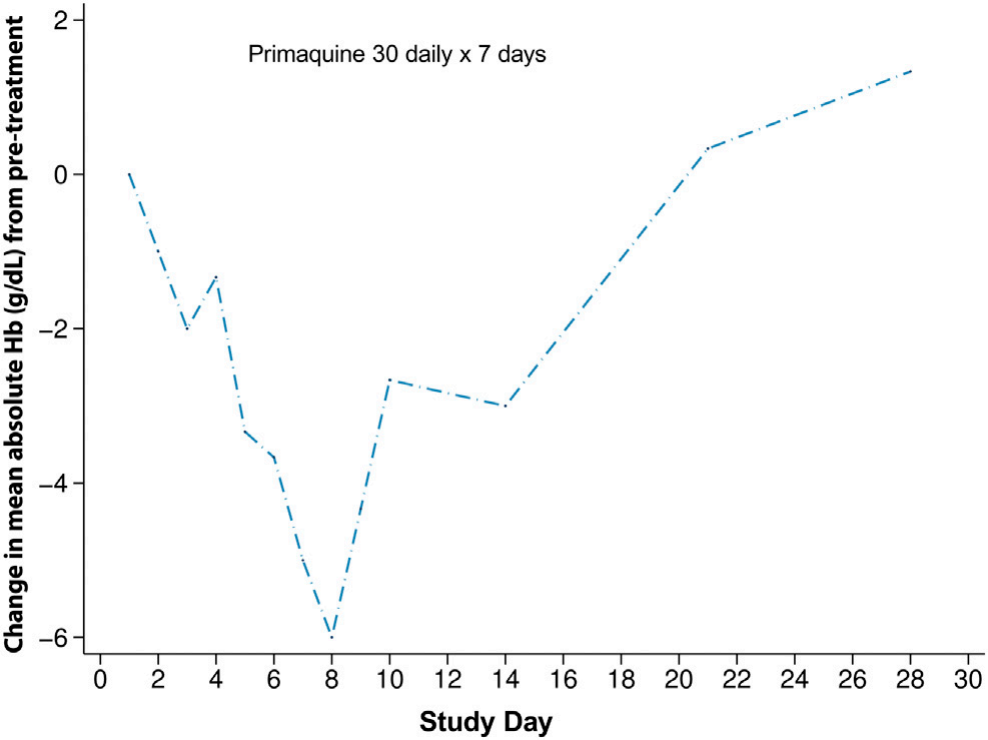
Haematologic changes in four G6PD deficient subjects with G6PD Asian variants receiving primaquine 30 mg daily for 7 days. This figure is replotted from published data from (Krudsood et al., 2006). The mean absolute haematocrit change was converted to haemoglobin and then a change from the mean pre-treatment value was plotted. This was done so that a comparison could be made to Figure 4.

Most of the studies that included detailed haematologic changes associated with G6PD deficiency were conducted in populations where G6PD Mahidol is predominant. In a study from 2006, four G6PD deficient patients with *P. vivax* malaria received primaquine 30 mg daily for 7 days. The mean haematocrit drops in these four patients were significantly greater than compared to elubaquine, another 8-aminoquinoline being assessed by Central Drug Research Institute (CDRI, India) at the time (Krudsood et al., 2006) (Figure 3). G6PD genotyping and the type of phenotypic test was not reported in this study.

**FIGURE 4:**
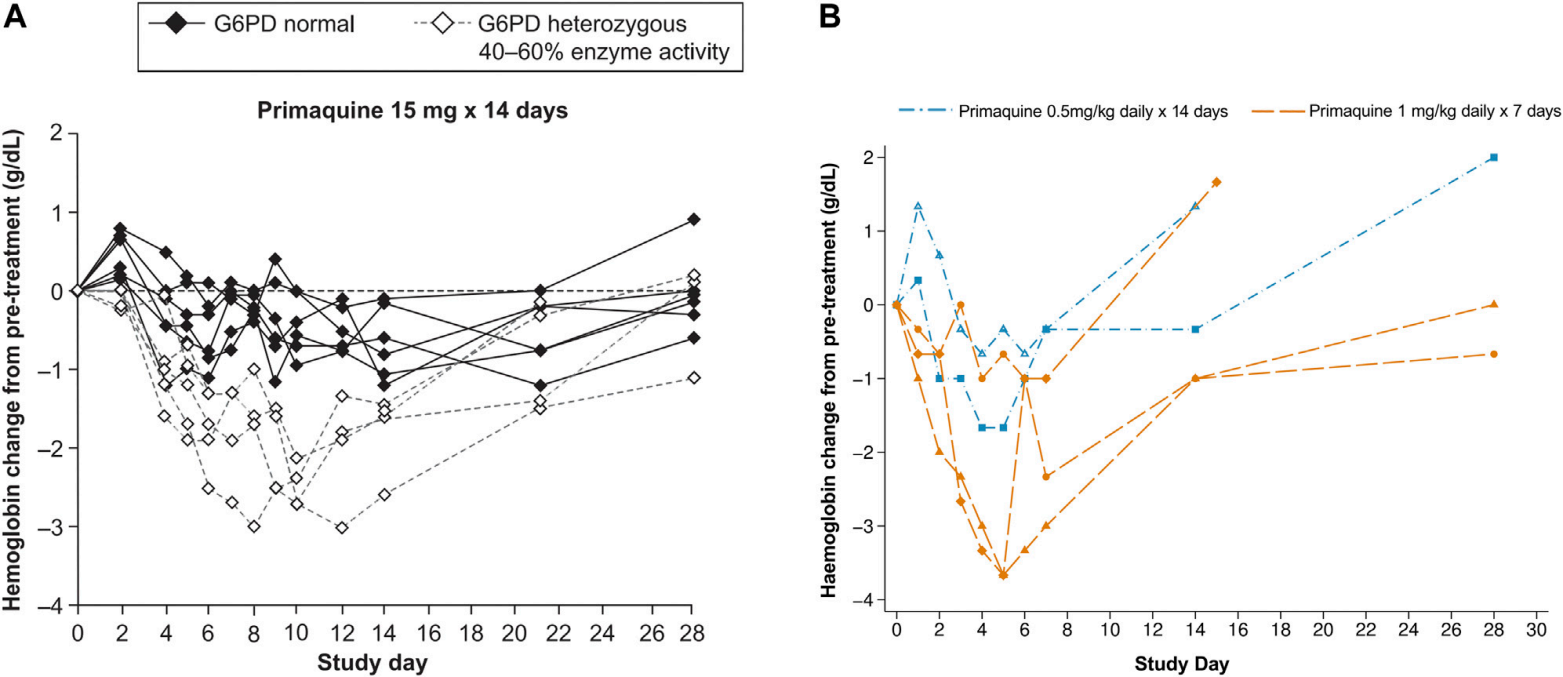
**(A, B)** Haematologic change in G6PD heterozygous females with intermediate G6PD activity (30–70%) receiving primaquine 15, 30, and 60 mg daily doses **(A)** Absolute haemoglobin changes in healthy G6PD Mahidol females with 40–60% G6PD activity during low dose primaquine treatment for *Plasmodium vivax* malaria. Figure 4A is taken from (Rueangweerayut et al., 2017), which is under a CC-BY license; raw data are not available. The dashed lines can be compared to Figure 3 and Figure 4B, however in this study, the population of G6PD heterozygous females was healthy and the primaquine doses were stopped in four of five subjects (on days 6 [*n* = 2], 9, and 10). **(B)** Absolute haematologic change in five G6PD*Mahidol heterozygous females with 30–70% G6PD activity during primaquine treatment for *Plasmodium vivax* malaria. Figure 4B is replotted from published data from (Chu et al., 2017). Haematocrit was converted to haemoglobin and the absolute change from pre-treatment for the individual subject was plotted. This was done so that a comparison could be made to Figure 4A. The subject represented by the diamond symbol received a blood transfusion.

Two contemporary studies have now added to the previously published data on the haematologic changes caused by primaquine in G6PD heterozygous females. In the first study, G6PD genotyping retrospectively identified Mahidol heterozygous females who tested normal using a qualitative rapid test, were enrolled in the study, and given primaquine for symptomatic *P. vivax* infection. These females had intermediate to normal G6PD activity and were eligible to receive routine treatment with primaquine. Most of these participants were asymptomatic or had mild symptoms. However, dose dependent haematocrit reductions were observed. When daily primaquine was given as 0.5 mg/kg (adult dose approximately 30 mg daily) for 14 days and 1 mg/kg (approximately 60 mg daily) for 7 days, greater haematocrit drops occurred with the high dose short course regimen in 33 G6PD heterozygous females (Chu et al., 2017) (Figure 4B). Two of them required a blood transfusion and only one of them reported symptoms; both completed the high dose short course primaquine regimen. In a second study, healthy G6PD heterozygous females were give a daily primaquine dose of 15 mg daily which was associated with haemoglobin drops (Rueangweerayut et al., 2017) (Figure 4A). Three of four subjects reached the pre-defined stopping criteria of an absolute haemoglobin drop ≥2.5 g/dl or fractional haematocrit drop of ≥7.5% from pre-treatment; one subject withdrew from the study. No clinical symptoms associated with haemolysis were noted. These two studies corroborate the widely variable haemolysis seen in the late 1950s during primaquine use in G6PD A- heterozygous females.

#### Weekly and Single Dose Primaquine

In Cambodia, tolerability and safety of weekly primaquine was assessed in a cohort of 75 patients where 18 of them had G6PD deficiency (17 Viangchan and one Canton variant). There was a severe adverse event in one G6PD deficient male (with Viangchan variant) who had taken ciprofloxacin and cimetidine (a weak CYP2D6 inhibitor) the previous day (Kheng et al., 2015). Ciprofloxacin induced AHA has been reported, but the AHA was potentially exacerbated by co-morbidities (Sansone et al., 2010). There is no consistent evidence that ciprofloxacin is a haemolytic agent (Youngster et al., 2010). While it appears the weekly primaquine dose is safe in G6PD deficient populations, drug-drug interactions should be considered. Interference with 8-aminoquinoline metabolism and drug increased exposure, and the presence of co-morbidities that affect bone marrow responses may need stronger consideration when analysing haemolytic risk and assessing haematologic recovery.

The tolerability and safety of primaquine single low dose (0.25 mg base/kg) combined with dihydroartemisinin-piperaquine (DHA-PPQ) given three times at monthly intervals was assessed in subjects with Mahidol, Chinese-4, Canton and Viangchan variants. Mean changes in the haemoglobin concentrations in both G6PD deficient (from −5.0 to −4.2%) and G6PD normal (from −1.7 to 0.3%) were small and clinically insignificant (Bancone et al., 2016).

#### Other Drugs

Tafenoquine, a new 8-aminoquinoline, also caused dose dependent haemolysis in G6PD heterozygous females with intermediate (40–60%) G6PD activity (Rueangweerayut et al., 2017). In Italy, a Filipino child with Vanua Lava mutation, a variant described in Indonesia and Malaysia (Satyagraha et al., 2015; Alina et al., 2020; Sulistyaningrum et al., 2020), developed AHA after paracetamol use (Minucci et al., 2011).

## Prediction of Drug-Induced Haemolysis

With renewed interest in 8-aminoquinolines and a substantial increase in the number of laboratories able to perform quantitative enzymatic tests, a question remains partially unanswered: is the enzymatic phenotype the best predictor of the haemolytic risk in individuals receiving a specific drug regimen? The short and general answer is that G6PD phenotype is the major factor influencing anti-oxidative response to challenges. However, as observed in most studies (*see* Figures 1B, 2), the time-course of haemolytic episodes is composed of a haemolytic phase followed by a recovery phase.

The G6PD deficient RBCs are the first to haemolyse when exposed to oxidative stress, therefore the number of G6PD depleted RBCs together with the concentration of the oxidative stressor in the blood are the best indicators of the magnitude of haemolysis observed in this phase. Following the oxidative insult, haemoglobin levels drop and the bone marrow responds by producing and releasing into circulation new cells (reticulocytes and young RBCs) that are less susceptible to haemolysis; this initiates a “recovery phase” where haemoglobin levels stabilize and then start to increase even with continuous exposure to the oxidant stressor. The number of new RBCs released into the peripheral circulation and their anti-oxidative status determine the change in haemoglobin drop course and the timing of instauration of a resistant phase. While the clinical severity of a haemolytic event depends mostly from the magnitude and speed of haemoglobin drops, the timing and capacity of bone marrow to respond by producing new G6PD replete RBCs is also crucial for haemoglobin recovery. Most haemolytic episodes are self-limited because reticulocytes and young RBCs provide sufficient anti-oxidant capacity. However, the Mediterranean variant and other G6PD variants associated with CNSHA (historically classified as Class I) have reticulocytes or young RBCs that are already G6PD depleted (Piomelli et al., 1968; Kaplan, 1974); individuals with these mutations are not able to establish a resistant phase and are at risk of continued haemolysis. Therefore, the overall clinical outcome depends on both the initial enzymatic phenotype and the genotype.

In addition to this, there are also several factors that have shown or are expected to have an impact on the haemolytic time course.

First of all, the accuracy of the technique used for phenotypic characterisation needs to be taken into consideration. The spectrophotometric assay is effectively used in normal practice as the reference test and it is very reliable in non-anaemic samples and in samples with a normal reticulocyte count. Nonetheless, the number of deficient RBCs (i.e. susceptible to haemolysis) is best assessed by cytologic techniques, especially when the read-out is done by flow-cytometer on large number of cells (>30,000) (Van Noorden and Vogels, 1985; Shah et al., 2012). Even in non-anaemic subjects, the flow-cytometric assay has detected different proportion of “G6PD-normal” RBCs in healthy A- hemizygous males compared to healthy Mahidol hemizygous males who showed comparable residual enzymatic activity by spectrophotometry (Kalnoky et al., 2018); potentially this could indicate a capacity of the flow-cytometric assay to capture subtle changes in RBCs phenotypes caused by different variants.

Secondly, different RBC conditions such as haemoglobinopathies (e.g., alpha and beta-thalassemia, haemoglobin structural variants) and membrane defects (e.g., ovalocytosis) influence the haemoglobin levels, the number of circulating reticulocytes (Bancone et al., 2017), and the intracellular oxidative state of erythrocytes (Vives Corrons et al., 1995; Margetis et al., 2007). These RBC conditions are particularly common in populations with G6PD deficiency (Lippi and Mattiuzzi, 2020). The impact of concomitant RBC conditions on the phenotypic characterisation of individuals with abnormal G6PD and on their haemolytic response to oxidative stress is not well characterised yet but might be substantial. Fever and infection, and the treatment of those infections with oxidative drugs can perturb the phenotypic G6PD activity steady state (Hersko and Vardy, 1967; Meyer, 1973; Berry and Melmed, 1977; Ahmad et al., 2018). These factors tend to cause haemolysis of older RBCs thus lowering the mean cell age within the RBC population and transiently shift the overall residual enzymatic activity towards higher levels.

Lastly, haemolytic factors that influence erythropoiesis are expected to impact on the time-course of haemoglobin by delaying recovery. Ineffective erythropoiesis associated with haemoglobinopathies, bone marrow suppression caused by infections, congenital dyserythropoietic diseases (Gangarossa et al., 1995), and iron or folate deficiency are all conditions yet to be explored in relation to drug-induced haemolysis.

## A Way Forward

In order to understand the haemolytic risk of unwell patients and healthy populations exposed to known oxidative antimalarials and other known or potentially haemolytic drugs (including first line antibiotics), prospective laboratory and clinical studies should be conducted.

From the laboratory perspective, a detailed characterization at steady-state of G6PD phenotypes associated to specific variants should include: 1) the assessment of residual enzymatic activity by spectrophotometric assay; 2) the proportion of normal and deficient RBCs by flow-cytometry; 3) the enzymatic phenotyping of reticulocyte (including ideally the dynamic of loss of protein enzymatic activity during erythrocyte maturation); 4) the assessment of RBCs intracellular oxidative stress alone and in the presence of concomitant haemoglobinopathies. In particular, the residual enzymatic activity in reticulocytes and young RBCs of most G6PD variants is unknown and might help to define a more clinically meaningful phenotype for variants historically classified in WHO class II and III.

From the clinical perspective, haemolytic challenges with common therapeutics (e.g., antimalarials, antibiotics) in heterozygous women with intermediate G6PD activity (30–70% of normal) would allow a safe analysis of the time-course of drug induced haemolysis (Beutler et al., 2007). In particular, together with the changes in haemoglobin levels and associated symptoms, assessments of intra-erythrocytic changes causing extra-vascular haemolysis (e.g., Heinz-bodies, GSH/GSSG, reduced deformability, etc.), timing and magnitude of bone-marrow response (including in individuals with iron and folate deficiencies, with ineffective erythropoiesis or bone marrow suppression by infection) and presence of co-morbidities would provide critical information on factors influencing haemolysis and recovery.

Additionally, pharmacodynamic correlations using *in vivo* drug concentrations and measures of haemolysis can inform on the relationship of drug metabolism (and CYP450 mutations) with haemolytic risk. In the example of primaquine, this would include an analysis of CYP2D6 genotypes and phenotypes (Baird et al., 2018).

Ultimately, comprehensive laboratory and clinical characterisation of haemoylsis and haematologic recovery will also contribute evidence to update the classification of G6PD variants in respect to haemolytic risk and allow clinicians and researchers to deploy oxidative drugs more safely.
