# Supplementary material for: G6PD Variants and Haemolytic Sensitivity to Primaquine and Other Drugs
Source: Front Pharmacol. 2021 Mar 15;12:638885. doi: 10.3389/fphar.2021.638885 (PMC8005603; doi:10.3389/fphar.2021.638885)
Supplement: Supplementary file 1 [file table1.docx]

**Supplementary Table 1**. Studies and case reports about drugs other than 8-aminoquinolines in G6PD deficient individuals.

| **Reference** | **Country (Ethnicity or Nationality)** | **G6PD variant** ^a^ **(tested or presumed)** | **Drug** | **Indication** | **Dose**^b^ **(oral unless specified)** | **Outcomes** | **N** |
| --- | --- | --- | --- | --- | --- | --- | --- |
| Degowin, Eppes et al. 1966 | USA (Afro-American) | Presumed A- | Dapsone and PQ | [Clinical trial in healthy volunteers] | 25-300mg daily x 21d | High Dapsone dose causes haemolysis; single 200mg dose does not cause haemolysis | 5 |
| Norden, Desforges et al. 1968 | USA | Presumed A- | Sulfonamide | Bacteraemia UTI | 500mg daily | No haemolysis in G6PDd adults; reticulocytosis in 3 of 4 G6PDd patients | 4 |
| Howell and Cook 1971 | West Indies | Presumed A- | Hycanthone | *Schistosoma mansoni* | 3mg/kg IM single dose | No haemolysis in G6PDd children patients | 6 |
| McCaffrey, Farid et al. 1972 | Egypt | B- | Ambilhar | *Schistosoma mansoni* | 25mg/kg daily x 7d | Compensated drug-induced haemolysis in G6PDd | 1 |
| Owusu 1972 | Ghana | Presumed A- | TMP-SMX (+chloramphenicol) | Typhoid fever | 80+400mg twice daily | AHA case report in G6PDd adult | 1 |
| Glader 1976 | USA (Afro-American and Caucasian) | Presumed A- and others | ASA | [Clinical trial in healthy volunteers] | 50mg/kg daily x 4d | No haemolysis in healthy G6PDd adults | 22 |
| Chugh, Singhal et al. 1977 | Northern India | Presumed Mediterranean | Chloroquine, ASA, Analgin, Chloramphenicol, Phenylbutazone or combinations | Various | Not specified | Acute renal failure | 30 |
| Corash, Spielberg et al. 1980 | Greece | Presumed Mediterranean | Vitamin E | Prevention of haemolysis | 800IU daily x >90d | Improved Hb, less retics, longer RBC half-life | 23 |
| Colonna 1981 | Algeria | Not characterized | ASA | Not specified | some normal some low (400mg in 6yo) | AHA in adult patients- possibly related to ASA | 40 |
| Bartsocas, Schulman et al. 1982 | Greece | Presumed Mediterranean | Paracetamol | Fever | 500mg single dose | AHA case report in G6PDd adult | 1 |
| Meloni, Forteleoni et al. 1982 | Italy | Mediterranean | Feprazone | Fever | 75mg suppository single dose | No haemolysis in G6PDd children patients | 30 |
| Sansone, Reali et al. 1984 | Italy | Presumed Mediterranean | Sodium metasolphan noramidipyrine | Fever | Not specified | AHA case report in G6PDd child (patient with *Salmonella typhi*) | 1 |
| Seeler and Luken 1984 | USA | Presumed A- | ASA (+ penicillin) | Acute rheumatic fever | 100mg/kg daily | AHA possibly induced by ARF before ASA treatment. | 1 |
| Markowitz and Saravolatz 1987 | USA | Presumed A- | TMP-SMX and vancomycin | *Staphylococcus aureus* | 320mg of TMP + 1600mg of SMX IV every 12 hr or vancomycin 1g IV every 12 hr | No haemolysis in G6PDd adult patients | 20 and 25 |
| Galun, Oren et al. 1987 | Israel | Presumed Mediterranean | Phenazopyridine | Urinary tract analgesic | 1200mg single dose | AHA case report G6PDd adult | 1 |
| Piperno, Muzio et al. 1987 | Italy | Presumed Mediterranean | Cimetropium bromide | Antispasmodic | 50mg thrice daily x 1w | No haemolysis in G6PDd healthy volunteers | 10 |
| Meloni, Forteleoni et al. 1989 | Italy | Mediterranean | ASA | Systematic arthritis | 100mg/kg x daily x 10d | AHA case report in G6PDd child | 1 |
| Khalifa, el-Alfy et al. 1989 | Egypt | Presumed Mediterranean | Desferrioxamine B | Haemolytic crisis | 500mg IV added to blood transfusion | Better hematologic outcome when added to transfusion in children with G6PD AHA | 24 |
| Choudhry, Ghafary et al. 1990 | Afghanistan | Presumed Mediterranean | CQ; PMQ; Chloramphenicol+ASA | Malaria | Not specified | AHA in G6PDd patients | 18 |
| Cottafava, Nieri et al. 1990 | Italy | Presumed Mediterranean | Paracetamol | Not specified | Not specified | No haemolysis in G6PDd children | 9 |
| Mehta, Singhal et al. 1990 | India | Presumed Mediterranean or Orissa | Vitamin C |  | Overdose, 3-4g within in 4-6 hr | AHA case report in G6PDd child | 1 |
| Gait 1990 | Worldwide |  | Nitrofurantoin |  | median 400mg | AHA case report G6PDd adults | 107 |
| Byrd and Gelber 1991 | USA | Presumed A- | Dapsone | Leprosy | 100mg daily | Hb decline >2g/dL (retrospective 100 patients) | 100 |
| Salazar, Carlos et al. 1991 | Philippines | Presumed Union | Halofantrine | Acute uncomplicated *P. falciparum* malaria | 1500mg | no haemolysis in G6PDd adults | 2 |
| Eldad, Neuman et al. 1991 | Israel | Presumed Mediterranean | Silver sulphadiazine | Open flame body burn | 1% topical twice daily | AHA case report in G6PDd adult | 1 |
| Rees, Kelsey et al. 1993 | UK (Nigerian) | Presumed A- | Vitamin C | Vitamin deficiency | 40g IV thrice daily, increased at 80g | AHA case report in G6PDd adult; G6PDd and HbAS, HIV+ | 1 |
| Reinke, Thomas et al. 1995 | USA (Afro-American) | Presumed A- | TMP-SMX | AIDS | 50mg/kg daily | AHA case report in G6PDd adult | 1 |
| Reinke, Thomas et al. 1995 | Worldwide | Not characterised | TMP-SMX or SMX alone |  | Several regimens | Review of AHA case reports in G6PDd | 15 |
| Kandil, al-Ghanem et al. 1996 | Kuwait | Presumed Mediterranean or A- | Henna (Lawsone) | Cosmetic | topical | NNJ in newborns with G6PDd | 15 |
| Haliotis, Tzortzinis et al. 1997 | Greece | Presumed Mediterranean | Tolfenamic | Antipyretic | Not specified | No haemolysis in G6PDd children | 10 |
| Chan 1997 | Hong Kong | Not characterized | TMP-SMX | Various | 480mg twice daily x 5d | No haemolysis in subjects aged >15 years treated with drug (retrospective analysis of hospital records) | 2163 (of whom ca.3% were expected to be G6PDd) |
| al-Rimawi, al-Sheyyab et al. 1999 | Jordan | Presumed Mediterranean | Desferrioxamine | Haemolytic crisis | 30-40mg/kg IV added to blood transfusion | Better hematologic outcome when added to transfusion in G6PDd children with AHA | 80 |
| Sood and Midha 2000 | Northern India | Presumed Mediterranean | Streptokinase (+ASA) | Thrombolytic | 1.5million units IV | AHA case report in G6PDd adult | 1 |
| Oliver, Coton et al. 2001 | France (Comorian) | A- | Propacetamol | Headache | Normal dose IV | AHA case report in G6PDd adult | 1 |
| Sklar 2002 | Singapore (Chinese) | Not characterized | Paracetamol | Headache | overdose 10-15g | AHA case report in G6PDd adult | 1 |
| Srivatsa, Bharti et al. 2003 | India | Presumed Mediterranean or Orissa | Nimesulide | Fever | 25 -100 mg single dose | AHA case report in G6PDd children | 2 |
| Vinzio, Andres et al. 2004 | France (Burkina Faso) | A- | Glibencamide + metformin | Diabetes | 5mg twice daily and 850 mg daily | AHA case report in G6PDd adult | 1 |
| Stalnikowicz, Amitai et al. 2004 | Israel | Not characterized | Butyl nitrite | Aphrodisiac | Not specified | AHA case report in G6PDd adults | 3 |
| Mandi, Witte et al. 2005 | Burkina Faso | A- | Methylene blue | Uncomplicated *P. falciparum* malaria | 780mg MB total | No haemolysis in G6PDd healthy adults | 74 |
| Meissner, Mandi et al. 2005 | Burkina Faso | A- | Methylene blue | Uncomplicated *P. falciparum* malaria | 12mg/kg total | No haemolysis in G6PDd children | 24 |
| Browning and Kruse 2005 | USA | Presumed A- | Rasburicase | Acute renal failure secondary to hyperuricemia | 22.5mg IV single dose | AHA case report in G6PDd adult | 1 |
| Krudsood, Wilairatana et al. 2006 | Thailand | Presumed Mahidol or Viangchan | Elubaquine | Anti-relapse *P. vivax* malaria | 25 mg daily x 7d | No haemolysis in G6PDd adults | 3 |
| Foltz, Dalal et al. 2006 | Canada (Filipino) | Presumed Union | Triapine | Metastatic renal cell  carcinoma | Not specified | AHA case report in G6PDd adult | 1 |
| Leslie, Mayan et al. 2007 | Afghanistan and Pakistan | Presumed Mediterranean | CD, SP or CQ | Malaria | 2.0 mg/kg C + 2.5 mg/kg D daily  Or 25mg S and 1.25 mg P on day0 or 250-mg base CQ over 3 days | AHA in G6PDd treated with CD | 1 |
| Fanello, Karema et al. 2008 | Rwanda | A- | CDA | Uncomplicated *P. falciparum* malaria | [2.0 mg/kg C + 2.5 mg/kg D daily + 4 mg/kg daily A] x 3d | Increased anaemia after treatment in G6PDd children | 44 |
| Piette, Taylor et al. 2008 | USA | Presumed A- and others | Dapsone gel | Acne | 5% Topical | No haemolysis in G6PDd children patients >12y | 62 |
| Stotland, Shalita et al. 2009 | USA and Canada | Presumed A- and others | Dapsone gel | Acne | 5% Topical | No haemolysis in G6PDd | 50 |
| Tiono, Dicko et al. 2009 | Burkina Faso | A- | CDA | Uncomplicated *P. falciparum* malaria | [2.0 mg/kg C + 2.5 mg/kg D daily + 4mg/kg daily A] x 3d | Increased % of G6PDd reaching Hb safety endpoint | 48 |
| Chisholm-Burns, Patanwala et al. 2010 | USA (Afro-American) | Presumed A- | TMP-SMX | UTI | 160/800 twice daily x 10d | AHA case report in G6PDd adult | 1 |
| Sansone, Rottensteiner et al. 2010 | Italy | Mediterranean | Ciprofloxacin | Fever | 500mg IV daily x 4d | AHA case report in G6PDd adult | 1 |
| Livshits, Hoffman et al. 2011 | USA (Afro-American) | Presumed A- | Mix of vitamins B and D complex, free amino acids, magnesium, and taurine |  | Not specified | AHA case report in G6PDd adult | 1 |
| Minucci, De Luca et al. 2011 | Italy (Filipino) | Vanua Lava | Paracetamol | Fever | Standard dose for 5y old | AHA case report in G6PDd child | 1 |
| Najafi, Van de Velde et al. 2011 | Belgium (different nationalities) | Not characterized | Paracetamol, ibuprofen, tramadol, sufentanil, and parecoxib | Post-surgery analgesia | standard dose | No haemolysis in G6PDd children patients | 10 |
| Van Malderen, Van Geertruyden et al. 2012 | Africa (7 countries) | A- | CDA | Uncomplicated *P. falciparum* malaria | [2.0 mg/kg daily C + 2.5 mg/kg daily D + 4mg/kg daily A] x 3d | More haemolysis in children CDA group | 26 |
| Sonbol, Yadav et al. 2013 | USA (Afro-American) | Presumed A- | Rasburicase (+ other drugs) | Refractory aggressive multiple myeloma | 6 mg | AHA case report in G6PDd adult | 1 |
| Muller, Mockenhaupt et al. 2013 | West Africa | A- | Methylene blue | Uncomplicated *P. falciparum* malaria | 15 mg/kg daily | No clinically relevant haemolysis in G6PDd children | 161 |
| Cheah, Lew et al. 2013 | Australia (Mauritian-Chinese) | Not characterized | Rasburicase | Tumour lysis syndrome | 6mg | AHA case report in G6PDd adult | 1 |
| Phillpotts, Tash et al. 2014 | UK (Pakistani) | Presumed Mediterranean | Paracetamol | Intentional overdose | Overdose 11 g (196 mg/kg) | AHA case report in G6PDd adult | 1 |
| Geraldino-Pardilla, Sung et al. 2014 | USA (Honduran) | A- | Pegloticase | Severe refractory gout | Normal dose | AHA case report in G6PDd adult | 1 |
| Huang, Chang et al. 2014 | China | Not characterized | Vitamin C |  | 2g every 4 hr IV x 2 days | AHA case report in G6PDd child | 1 |
| Biscaglia, Ferri et al. 2015 | Italy | Mediterranean and Seattle | ASA | Percutaneous Coronary intervention | 100mg daily | No haemolysis in G6PDd adult patients | 5 |
| Owusu, Asante et al. 2015 | Ghana | Presumed A- | Sulfadoxine-Pyrimethamine | Malaria – Intermittent Presumptive Treatment | 1500mg S + 75 mg P | No haemolysis in G6PDd adult patients | 291 |
| Poirot, Vittinghoff et al. 2015 | Tanzania | A- | CD, Mefloquine and SP | Malaria – Intermittent Presumptive Treatment in infants | 250mg S, 12.5mg P, 15mg C + 18.75mg D, 125mg M | Greater Hb drop in G6PDd children in CD group | 28 |
| Reeves, Saum et al. 2016 | USA (Afro-American) | Presumed A- | Rasburicase | Multiple myeloma | 6mg IV | AHA case report in G6PDd adult | 1 |
| Murphy and Grossman 2016 | USA (Egyptian) | Presumed Mediterranean | Dapsone | *Pneumocystis jirovecii* prophylaxis in glioblastoma patient | 100mg daily | AHA case report in G6PDd adult | 1 |
| Rickner, Cao et al. 2017 | USA | Not characterized | Paracetamol + Amoxicillin | Not specified | overdose 150mg/kg P, 15mg/kg A | AHA case report in G6PDd adult | 1 |
| Sharma, Srinivasaraghavan et al. 2017 | India | Not characterized | Norfloxacin and metronidazole | Fever | Not specified | AHA case report in G6PDd child | 1 |
| Kheir, Gaber et al. 2017 | Sudan | Presumed A- | Henna (Lawsone) | Cosmetic | Topical | AHA case report in G6PDd child | 1 |
| Lai, Huang et al. 2017 | China | Not characterized | Sodium dimercaptosulphonate therapy | Wilson's disease | 0.75 to 1g daily x 3-7d | AHA case report in G6PDd adult | 2 |
| Feghaly, Al Hout et al. 2017 | Worldwide | Not characterized | ASA | Percutaneous Coronary intervention | Not specified | No haemolysis in G6PDd adult patients | 4 (excluding cases from Biscaglia, Ferri et al. 2015) |
| Quinn, Gerber et al. 2017 | USA (Afro-American) | Presumed A- | Vitamin C | Rheumatoid arthritis | 75g IV single dose | AHA case report in G6PDd adult | 1 |
| Wu, Fu et al. 2018 | China | Not characterized | Alteplase | Ischaemic stroke | 0.9 mg/kg (max 90 mg) | No haemolysis in G6PDd adult patients | 20 |
| Rees, Strach et al. 2018 | Australia | Not characterized | Vitamin C |  | 30g IV single dose | AHA case report in G6PDd adult | 1 |
| Minshar, Osman-Malik et al. 2019 | Australia (Afro-American) | Presumed A- | Pegloticase | Gout | 8mg IV | AHA case report in G6PDd adult | 1 |
| Chang, Yang et al. 2018 | Taiwan | Presumed Canton or Kaiping | Anidulafungin (+ Clindamycin) | *Pneumocystis jirovecii* prophylaxis in HIV patient | 200mg IV loading then 100 mg IV daily A, 600 mg IV every 8 hr C | No haemolysis in G6PDd adult patient | 1 |
| Chung, Hsu et al. 2019 | Taiwan | Presumed Canton or Kaiping | Doxorubicin, cyclophosphamide, docetaxel, trastuzumab | Breast cancer | Not specified | No haemolysis in G6PDd adult patient | 1 |
| Lu and Chen 2020 | Taiwan | Presumed Canton or Kaiping | TMP-SMX | *Pneumocystis jirovecii* pneumonia | 240mg/1200mg IV every 8 hr x 21d | No haemolysis in G6PDd adult patient | 1 |
| Chen, Li et al. 2020 | China | Not characterized | ASA | Ischaemic stroke | 100mg daily x 3 months | No haemolysis in G6PDd adult patient; higher risk of death in G6PDd | 80 |

^a^ When data available, the most common variant was presumed. For Algeria and other countries with extremely high G6PD molecular heterogeneity, no presumed variant was listed.

^b^ The doses reported in table reflect what was reported in the manuscript and may not be complete.

**Abbreviations**

AHA= Acute Haemolytic Anaemia; ARF= Acute renal failure, ASA= Acetylsalicylic Acid; PMQ=Primaquine; CDA= Chlorproguanil-Dapsone Artesunate; CD= Chlorproguanil-Dapsone; CQ= Chloroquine; SP= Sulphadoxine-Pyrimethamine; d=day; G6PDd= G6PD deficiency; Hb= haemoglobin, HbAS= sickle cell trait; NNJ= neonatal jaundice; TMP-SMX= Trimethoprim/sulfamethoxazole

**References**

al-Rimawi, H. S., M. al-Sheyyab, A. Batieha, H. el-Shanti and F. Abuekteish (1999). "Effect of desferrioxamine in acute haemolytic anaemia of glucose-6-phosphate dehydrogenase deficiency." Acta Haematol **101**(3): 145-148.

Bartsocas, C. S., J. D. Schulman and L. Corash (1982). "Can acetaminophen cause hemolysis in G6PD deficiency?" Acta Haematol **67**(3): 228.

Biscaglia, S., A. Ferri, R. Pavasini, G. Campo and R. Ferrari (2015). "Dual Antiplatelet Therapy in Patients with Glucose-6-Phosphate Dehydrogenase Deficiency undergoing PCI with Drug-Eluting Stents." J Atheroscler Thromb **22**(5): 535-541.

Browning, L. A. and J. A. Kruse (2005). "Hemolysis and methemoglobinemia secondary to rasburicase administration." Ann Pharmacother **39**(11): 1932-1935.

Byrd, S. R. and R. H. Gelber (1991). "Effect of dapsone on haemoglobin concentration in patients with leprosy." Lepr Rev **62**(2): 171-178.

Chan, T. Y. (1997). "Co-trimoxazole-induced severe haemolysis: the experience of a large general hospital in Hong Kong." Pharmacoepidemiol Drug Saf **6**(2): 89-92.

Chang, H. C., W. T. Yang and T. C. Chen (2018). "Pneumocystis jirovecii pneumonia in a human immunodeficiency virus-infected patient with G6PD deficiency-successful treatment with anidulafungin." Eur Rev Med Pharmacol Sci **22**(24): 8961-8964.

Cheah, C. Y., T. E. Lew, J. F. Seymour and K. Burbury (2013). "Rasburicase causing severe oxidative hemolysis and methemoglobinemia in a patient with previously unrecognized glucose-6-phosphate dehydrogenase deficiency." Acta Haematol **130**(4): 254-259.

Chen, Y., J. Li, Z. Ou, Y. Zhang, Z. Liang, W. Deng, W. Huang, Z. Wu, H. Jiang, Q. Liu, F. Ouyang, S. Xing and J. Zeng (2020). "Safety and efficacy of low-dose aspirin in ischemic stroke patients with different G6PD conditions." Int J Stroke: 1747493020950903.

Chisholm-Burns, M. A., A. E. Patanwala and C. A. Spivey (2010). "Aseptic meningitis, hemolytic anemia, hepatitis, and orthostatic hypotension in a patient treated with trimethoprim-sulfamethoxazole." Am J Health Syst Pharm **67**(2): 123-127.

Choudhry, V. P., A. Ghafary, M. Zaher, M. A. Qureshi, I. Fazel and R. Ghani (1990). "Drug-induced haemolysis and renal failure in children with glucose-6-phosphate dehydrogenase deficiency in Afghanistan." Ann Trop Paediatr **10**(4): 335-338.

Chugh, K. S., P. C. Singhal, B. K. Sharma, A. C. Mahakur, Y. Pal, B. N. Datta and K. C. Das (1977). "Acute renal failure due to intravascular hemolysis in the North Indian patients." Am J Med Sci **274**(2): 139-146.

Chung, W. P., Y. T. Hsu, Y. P. Chen and H. P. Hsu (2019). "Treatment of a patient with breast cancer and glucose 6-phosphate dehydrogenase deficiency: A case report." Medicine (Baltimore) **98**(13): e14987.

Colonna, P. (1981). "Aspirin and glucose-6-phosphate dehydrogenase deficiency." Br Med J (Clin Res Ed) **283**(6300): 1189.

Corash, L., S. Spielberg, C. Bartsocas, L. Boxer, R. Steinherz, M. Sheetz, M. Egan, J. Schlessleman and J. D. Schulman (1980). "Reduced chronic hemolysis during high-dose vitamin E administration in Mediterranean-type glucose-6-phosphate dehydrogenase deficiency." N Engl J Med **303**(8): 416-420.

Cottafava, F., S. Nieri, G. Franzone, M. Sanguinetti, L. Bertolazzi and G. Ravera (1990). "[Double-blind controlled comparison of placebo and paracetamol in patients with G-6-PD deficiency]." Pediatr Med Chir **12**(6): 631-637.

Degowin, R. L., R. B. Eppes, R. D. Powell and P. E. Carson (1966). "The haemolytic effects of diaphenylsulfone (DDS) in normal subjects and in those with glucose-6-phosphate-dehydrogenase deficiency." Bull World Health Organ **35**(2): 165-179.

Eldad, A., A. Neuman, A. Weinberg, P. Benmeir, M. Rotem and M. R. Wexler (1991). "Silver sulphadiazine-induced haemolytic anaemia in a glucose-6-phosphate dehydrogenase-deficient burn patient." Burns **17**(5): 430-432.

Fanello, C. I., C. Karema, P. Avellino, G. Bancone, A. Uwimana, S. J. Lee, U. d'Alessandro and D. Modiano (2008). "High risk of severe anaemia after chlorproguanil-dapsone+artesunate antimalarial treatment in patients with G6PD (A-) deficiency." PLoS One **3**(12): e4031.

Feghaly, J., A. R. Al Hout and M. Mercieca Balbi (2017). "Aspirin safety in glucose-6-phosphate dehydrogenase deficiency patients with acute coronary syndrome undergoing percutaneous coronary intervention." BMJ Case Rep **2017**.

Foltz, L. M., B. I. Dalal, L. D. Wadsworth, R. Broady, K. Chi, E. Eisenhauer, K. Kobayashi and C. Kollmannsburger (2006). "Recognition and management of methemoglobinemia and hemolysis in a G6PD-deficient patient on experimental anticancer drug Triapine." Am J Hematol **81**(3): 210-211.

Gait, J. E. (1990). "Hemolytic reactions to nitrofurantoin in patients with glucose-6-phosphate dehydrogenase deficiency: theory and practice." DICP **24**(12): 1210-1213.

Galun, E., R. Oren, M. Glikson, M. Friedlander and A. Heyman (1987). "Phenazopyridine-induced hemolytic anemia in G-6-PD deficiency." Drug Intell Clin Pharm **21**(11): 921-922.

Geraldino-Pardilla, L., D. Sung, J. Z. Xu, M. Shirazi, E. A. Hod and R. O. Francis (2014). "Methaemoglobinaemia and haemolysis following pegloticase infusion for refractory gout in a patient with a falsely negative glucose-6-phosphate dehydrogenase deficiency result." Rheumatology (Oxford) **53**(12): 2310-2311.

Glader, B. E. (1976). "Evaluation of the hemolytic role of aspirin in glucose-6-phosphate dehydrogenase deficiency." J Pediatr **89**(6): 1027-1028.

Haliotis, F. A., A. A. Tzortzinis and D. A. Papanastasiou (1997). "Use of tolfenamic acid in febrile children with and without glucose-6-phosphate dehydrogenase deficiency." Int J Clin Pharmacol Ther **35**(3): 103-106.

Howell, S. B. and J. A. Cook (1971). "Treatment of schistosomiasis mansoni with hycanthone in glucose-6-phosphate dehydrogenase deficiency in St. Lucia." Trans R Soc Trop Med Hyg **65**(3): 331-333.

Huang, Y. C., T. K. Chang, Y. C. Fu and S. L. Jan (2014). "C for colored urine: acute hemolysis induced by high-dose ascorbic acid." Clin Toxicol (Phila) **52**(9): 984.

Kandil, H. H., M. M. al-Ghanem, M. A. Sarwat and F. S. al-Thallab (1996). "Henna (Lawsonia inermis Linn.) inducing haemolysis among G6PD-deficient newborns. A new clinical observation." Ann Trop Paediatr **16**(4): 287-291.

Khalifa, A. S., M. S. el-Alfy, G. Mokhtar, A. A. Fakeir, M. A. Khazbak, F. el-Baz and M. el-Kholy (1989). "Effect of desferrioxamine B on hemolysis in glucose-6-phosphate dehydrogenase deficiency." Acta Haematol **82**(3): 113-116.

Kheir, A., I. Gaber, S. Gafer and W. Ahmed (2017). "Life-threatening haemolysis induced by henna in a Sudanese child with glucose-6-phosphate dehydrogenase deficiency." East Mediterr Health J **23**(1): 28-30.

Krudsood, S., P. Wilairatana, N. Tangpukdee, K. Chalermrut, S. Srivilairit, V. Thanachartwet, S. Muangnoicharoen, N. Luplertlop, G. M. Brittenham and S. Looareesuwan (2006). "Safety and tolerability of elubaquine (bulaquine, CDRI 80/53) for treatment of Plasmidium vivax malaria in Thailand." Korean J Parasitol **44**(3): 221-228.

Lai, S., Y. Q. Huang, A. Q. Liu and H. W. Wu (2017). "Haemolysis during sodium dimercaptosulphonate therapy for Wilson's disease in G6PD-deficient patients: First report of two cases." J Clin Pharm Ther **42**(6): 783-785.

Leslie, T., M. I. Mayan, M. A. Hasan, M. H. Safi, E. Klinkenberg, C. J. Whitty and M. Rowland (2007). "Sulfadoxine-pyrimethamine, chlorproguanil-dapsone, or chloroquine for the treatment of Plasmodium vivax malaria in Afghanistan and Pakistan: a randomized controlled trial." JAMA **297**(20): 2201-2209.

Livshits, Z., R. S. Hoffman, K. B. Hymes and L. S. Nelson (2011). "If vitamins could kill: massive hemolysis following naturopathic vitamin infusion." J Med Toxicol **7**(3): 224-226.

Lu, Y. W. and T. C. Chen (2020). "Use of trimethoprim-sulfamethoxazole in a patient with G6PD deficiency for treating Pneumocystis jirovecii pneumonia without haemolysis: Case report and literature review." J Clin Pharm Ther **45**(6): 1483-1485.

Mandi, G., S. Witte, P. Meissner, B. Coulibaly, U. Mansmann, J. Rengelshausen, W. Schiek, A. Jahn, M. Sanon, K. Wust, I. Walter-Sack, G. Mikus, J. Burhenne, K. D. Riedel, H. Schirmer, B. Kouyate and O. Muller (2005). "Safety of the combination of chloroquine and methylene blue in healthy adult men with G6PD deficiency from rural Burkina Faso." Trop Med Int Health **10**(1): 32-38.

Markowitz, N. and L. D. Saravolatz (1987). "Use of trimethoprim-sulfamethoxazole in a glucose-6-phosphate dehydrogenase-deficient population." Rev Infect Dis **9 Suppl 2**: S218-229.

McCaffrey, R. P., Z. Farid and D. C. Kent (1972). "Acute haemolysis with Ambilhar treatment in glucose-6-phosphate dehydrogenase deficiency." Trans R Soc Trop Med Hyg **66**(5): 795-797.

Mehta, J. B., S. B. Singhal and B. C. Mehta (1990). "Ascorbic-acid-induced haemolysis in G-6-PD deficiency." Lancet **336**(8720): 944.

Meissner, P. E., G. Mandi, S. Witte, B. Coulibaly, U. Mansmann, J. Rengelshausen, W. Schiek, A. Jahn, M. Sanon, T. Tapsoba, I. Walter-Sack, G. Mikus, J. Burhenne, K. D. Riedel, H. Schirmer, B. Kouyate and O. Muller (2005). "Safety of the methylene blue plus chloroquine combination in the treatment of uncomplicated falciparum malaria in young children of Burkina Faso [ISRCTN27290841]." Malar J **4**: 45.

Meloni, T., G. Forteleoni, A. Ogana and V. Franca (1989). "Aspirin-induced acute haemolytic anaemia in glucose-6-phosphate dehydrogenase-deficient children with systemic arthritis." Acta Haematol **81**(4): 208-209.

Meloni, T., G. Forteleoni, M. Serra, S. Daniotti, L. Negri, P. Giuntini, L. Canepa and G. F. Gaetani (1982). "Feprazone: absence of hemolytic effects in glucose-6-phosphate dehydrogenase-deficient subjects." J Clin Pharmacol **22**(8-9): 418-420.

Minshar, M. A., Y. Osman-Malik and Z. Y. Bhat (2019). "Pegloticase-Associated Hemolysis." Am J Ther **26**(5): e622-e624.

Minucci, A., D. De Luca, E. Torti, P. Concolino, P. Maurizi, B. Giardina, C. Zuppi and E. Capoluongo (2011). "Acute haemolytic crisis due to concomitant presence of infection and possible altered acetaminophen catabolism in a Philipino child carrying the G6PD-Vanua Lava mutation." Ann Clin Biochem **48**(Pt 3): 282-285.

Muller, O., F. P. Mockenhaupt, B. Marks, P. Meissner, B. Coulibaly, R. Kuhnert, H. Buchner, R. H. Schirmer, I. Walter-Sack, A. Sie and U. Mansmann (2013). "Haemolysis risk in methylene blue treatment of G6PD-sufficient and G6PD-deficient West-African children with uncomplicated falciparum malaria: a synopsis of four RCTs." Pharmacoepidemiol Drug Saf **22**(4): 376-385.

Murphy, A. G. and S. A. Grossman (2016). "Acute hemolysis in a patient with a newly diagnosed glioblastoma." CNS Oncol **5**(3): 125-129.

Najafi, N., A. Van de Velde and J. Poelaert (2011). "Potential risks of hemolysis after short-term administration of analgesics in children with glucose-6-phosphate dehydrogenase deficiency." J Pediatr **159**(6): 1023-1028.

Norden, C. W., J. F. Desforges and E. H. Kass (1968). "Hemolytic effect of sulfonamides in patients with erythrocytes deficient in glucose-6-phosphate dehydrogenase." N Engl J Med **279**(1): 30-31.

Oliver, M., T. Coton, C. Badens, C. Dehan, D. Lena-Russo and J. L. Moalic (2001). "Homozygous G6PD deficiency and propacetamol induced hemolysis." Haematologica **86**(9): 987-988.

Owusu, R., K. P. Asante, E. Mahama, E. Awini, T. Anyorigiya, D. Dosoo, A. Amu, G. Jakpa, E. Ofei, S. Segbaya, A. R. Oduro, M. Gyapong, A. Hodgson, C. Bart-Plange and S. Owusu-Agyei (2015). "Glucose-6-Phosphate Dehydrogenase Deficiency and Haemoglobin Drop after Sulphadoxine-Pyrimethamine Use for Intermittent Preventive Treatment of Malaria during Pregnancy in Ghana - A Cohort Study." PLoS One **10**(9): e0136828.

Owusu, S. K. (1972). "Acute haemolysis complicating co-trimoxazole therapy for typhoid fever in a patient with G.-6-P.D. deficiency." Lancet **2**(7781): 819.

Phillpotts, S., E. Tash and S. Sen (2014). "Glucose-6-phosphate dehydrogenase deficiency: an unusual cause of acute jaundice after paracetamol overdose." Eur J Haematol **93**(5): 446-448.

Piette, W. W., S. Taylor, D. Pariser, M. Jarratt, P. Sheth and D. Wilson (2008). "Hematologic safety of dapsone gel, 5%, for topical treatment of acne vulgaris." Arch Dermatol **144**(12): 1564-1570.

Piperno, A., F. Muzio, B. P. Imbimbo, S. Manoussakis, S. Daniotti and G. Fiorelli (1987). "Cimetropium bromide, a new antispasmodic agent, has no hemolytic effects in humans." Int J Clin Pharmacol Ther Toxicol **25**(8): 464-467.

Poirot, E., E. Vittinghoff, D. Ishengoma, M. Alifrangis, I. Carneiro, R. Hashim, V. Baraka, J. Mosha, S. Gesase, D. Chandramohan and R. Gosling (2015). "Risks of Hemolysis in Glucose-6-Phosphate Dehydrogenase Deficient Infants Exposed to Chlorproguanil-Dapsone, Mefloquine and Sulfadoxine-Pyrimethamine as Part of Intermittent Presumptive Treatment of Malaria in Infants." PLoS One **10**(11): e0142414.

Quinn, J., B. Gerber, R. Fouche, K. Kenyon, Z. Blom and P. Muthukanagaraj (2017). "Effect of High-Dose Vitamin C Infusion in a Glucose-6-Phosphate Dehydrogenase-Deficient Patient." Case Rep Med **2017**: 5202606.

Rees, D. C., H. Kelsey and J. D. Richards (1993). "Acute haemolysis induced by high dose ascorbic acid in glucose-6-phosphate dehydrogenase deficiency." BMJ **306**(6881): 841-842.

Rees, M. J., M. C. Strach, K. Burbury and K. A. Phillips (2018). "Massive oxidative haemolysis and renal failure caused by high dose vitamin C." Med J Aust **209**(6): 248-249.

Reeves, D. J., L. M. Saum and R. Birhiray (2016). "I.V. ascorbic acid for treatment of apparent rasburicase-induced methemoglobinemia in a patient with acute kidney injury and assumed glucose-6-phosphate dehydrogenase deficiency." Am J Health Syst Pharm **73**(9): e238-242.

Reinke, C. M., J. K. Thomas and A. H. Graves (1995). "Apparent hemolysis in an AIDS patient receiving trimethoprim/sulfamethoxazole: case report and literature review." J Pharm Technol **11**(6): 256-262; quiz 293-255.

Rickner, S. S., D. Cao and S. E. Simpson (2017). "Hemolytic crisis following acetaminophen overdose in a patient with G6PD deficiency." Clin Toxicol (Phila) **55**(1): 74-75.

Salazar, N. P., C. C. Carlos, D. G. Bustos, B. P. Quiambao, M. C. Saniel and M. N. Santos (1991). "Halofantrine in the treatment of acute uncomplicated falciparum malaria in the Philippines." Southeast Asian J Trop Med Public Health **22**(3): 386-392.

Sansone, G., S. Reali, R. Sansone and F. Allegranza (1984). "Acute hemolytic anemia induced by a pyrazolonic drug in a child with glucose-6-phosphate dehydrogenase deficiency." Acta Haematol **72**(4): 285-287.

Sansone, S., J. Rottensteiner, J. Stocker, C. Rosanelli and C. J. Wiedermann (2010). "Ciprofloxacin-induced acute haemolytic anaemia in a patient with glucose-6-phosphate dehydrogenase Mediterranean deficiency: a case report." Ann Hematol **89**(9): 935-937.

Seeler, R. A. and J. A. Luken (1984). "Hemolysis in ARF in a G6PD-deficient patient: case 2." Pediatr Cardiol **5**(4): 328-329.

Sharma, S., R. Srinivasaraghavan and S. Krishnamurthy (2017). "Central Nervous System Symptoms Due to Transient Methemoglobinemia in a Child With G6PD Deficiency." J Pediatr Hematol Oncol **39**(1): e27-e28.

Sklar, G. E. (2002). "Hemolysis as a potential complication of acetaminophen overdose in a patient with glucose-6-phosphate dehydrogenase deficiency." Pharmacotherapy **22**(5): 656-658.

Sonbol, M. B., H. Yadav, R. Vaidya, V. Rana and T. E. Witzig (2013). "Methemoglobinemia and hemolysis in a patient with G6PD deficiency treated with rasburicase." Am J Hematol **88**(2): 152-154.

Sood, N. and V. Midha (2000). "Streptokinase-induced jaundice due to hemolysis in a G-6PD-deficient patient." Am J Gastroenterol **95**(1): 312-313.

Srivatsa, A., B. Bharti and S. C. Singhi (2003). "Does nimesulide induce haemolysis in glucose-6-phosphate dehydrogenase deficiency?" Acta Paediatr **92**(5): 637-638.

Stalnikowicz, R., Y. Amitai and Y. Bentur (2004). "Aphrodisiac drug-induced hemolysis." J Toxicol Clin Toxicol **42**(3): 313-316.

Stotland, M., A. R. Shalita and R. F. Kissling (2009). "Dapsone 5% gel: a review of its efficacy and safety in the treatment of acne vulgaris." Am J Clin Dermatol **10**(4): 221-227.

Tiono, A. B., A. Dicko, D. A. Ndububa, T. Agbenyega, S. Pitmang, J. Awobusuyi, A. Pamba, S. Duparc, L. E. Goh, E. Harrell, N. Carter, S. A. Ward, B. Greenwood and P. A. Winstanley (2009). "Chlorproguanil-dapsone-artesunate versus chlorproguanil-dapsone: a randomized, double-blind, phase III trial in African children, adolescents, and adults with uncomplicated Plasmodium falciparum malaria." Am J Trop Med Hyg **81**(6): 969-978.

Van Malderen, C., J. P. Van Geertruyden, S. Machevo, R. Gonzalez, Q. Bassat, A. Talisuna, A. Yeka, C. Nabasumba, P. Piola, A. Daniel, E. Turyakira, P. Forret, C. Van Overmeir, H. van Loen, A. Robert and D. A. U (2012). "Glucose-6-phosphate dehydrogenase deficiency, chlorproguanil-dapsone with artesunate and post-treatment haemolysis in African children treated for uncomplicated malaria." Malar J **11**: 139.

Vinzio, S., E. Andres, A. E. Perrin, J. L. Schlienger and B. Goichot (2004). "Glibenclamide-induced acute haemolytic anaemia revealing a G6PD-deficiency." Diabetes Res Clin Pract **64**(3): 181-183.

Wu, X., R. Fu, Y. Tang, L. Shi, X. Rong, J. Guo, J. Li and Q. Shen (2018). "Intravenous Thrombolysis for Stroke Patients with G6PD Deficiency." J Stroke Cerebrovasc Dis **27**(7): 2026-2031.
